# Chemical labelling of active serum thioester proteins for quantification

**DOI:** 10.1016/j.imbio.2011.07.021

**Published:** 2012-02

**Authors:** Lotta Holm, Gareth L. Ackland, Mark R. Edwards, Ross A. Breckenridge, Robert B. Sim, John Offer

**Affiliations:** a Division of Physical Biochemistry, MRC National Institute for Medical Research, The Ridgeway, Mill Hill, London NW7 1AA, UK; b Department of Medicine, University College London, London, UK; c Centre for Anaesthesia, Critical Care and Pain Medicine, University College London Hospitals, University College London, London, UK; d Department of Pharmacology, University of Oxford, Mansfield Road, Oxford OX1 3QT, UK

**Keywords:** C3, complement component 3, C3(H_2_O), C3 with hydrolysed thioester, C3(NH_2_NH_2_), hydrazine labelled C3, C3(bio), C3 labelled with biotin–PEG_4_–hydrazide, C3(N), C3 labelled with nucleophile at thioester bond, fI, complement factor I, fH, complement factor H, EACA, epsilon amino caproic acid, α-2M, α-2 macroglobulin, PEG-plasma, plasma precipitated by PEG 3350, SA, streptavidin, HRP, horseradish peroxidase, Acyl transfer, Human complement C3, Patient C3 detection, Thioester reactive probes

## Abstract

The complement serum proteins C3 and C4 and the protease inhibitor α-2 macroglobulin are all members of the C3/α-2M thioester protein family, an evolutionarily ancient and conserved family that contains an intrachain thioester bond. The chemistry of the thioester bond is a key to the function of the thioester proteins. All these proteins function by covalently linking to their target by acyl transfer of the protein via the thioester moiety. We show that the signature thioester bond can be targeted with nucleophiles linked to a bioreporter molecule, site-specifically modifying the whole, intact thioester protein. Conditions were optimised to label selectively and efficiently pull-down unprocessed thioester-containing proteins from serum. We demonstrated pull-down of full-length C3, α-2M and C4 from sera in high salt, using a biotinylated nucleophile and streptavidin-coated resin, confirmed by MALDI-TOF MS identification of the gel bands. The potential for the development of a quantitative method for measuring active C3 in serum was investigated in patient sera pre and post operation. Quantifying active C3 in clinical assays using current methods is difficult. Methods based on antibody detection (e.g. nephelometry) do not distinguish between active C3 and inactive breakdown products. C3-specific haemolytic assays can be used, but these require use of relatively unstable reagents. The current work represents a promising robust, enzyme- and antibody-free chemical method for detecting active thioester proteins in blood, plasma or serum.

## Introduction

The C3 and C4 thioester proteins of the complement pathway and the protease inhibitor α-2 macroglobulin (α-2M) form the first line of defence of the innate immune system against pathogens. Following limited proteolysis, C3 and C4 attach covalently to pathogen surfaces by acyl transfer via their thioester functionality and this irreversible attachment is central to pathogen recognition and clearance. α-2M interacts with exogenous proteases, and binds covalently to them, inhibiting their activity. In contrast to their protective effects, inappropriate or excessive activation of C3 is involved in many autoimmune, degenerative and inflammatory diseases. There is therefore great interest in techniques that can monitor the concentration of the active forms of these proteins as potential indicators of disease progression.

**Fig. 1:**
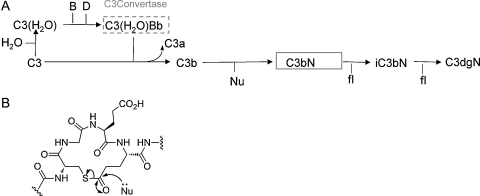
Reactivity of the unactivated thioester of complement protein C3. (A) The tick over event of the alternative pathway. C3(H_2_O) is formed by the slow hydrolysis of C3. Interaction between factor B and C3(H_2_O) forms an active C3 convertase complex that can generate more C3b molecules by cleavage of the C3a domain from C3, forming a positive feedback loop. C3b has a metastable thioester group that can react rapidly with nucleophiles such as the hydroxyl groups on carbohydrates or amines on proteins. (B) A generalised reaction mechanism for the reaction of a nucleophile with the thioester of full-length C3 (tick over event). This gives a route for the modification of the whole C3 protein without activation. The fifteen-membered intramolecular thiolactone ring is the defining feature of the C3/α-2M thioester protein family, an evolutionary ancient family of proteins that all function by forming covalent linkage of the protein to a target via the thioester bond. The thioester moiety is formed by bridging the cysteine and glutamine side chains with a β-cysteinyl-γ-glutamyl bond.

The site-specific labelling of proteins within a complex biological mixture like serum or plasma requires chemoselective reaction of the label with a unique site of a protein under near physiological conditions and in the presence of a vast array of other biomolecules. Some chemoselective reactions have been harnessed for activity based labelling, contributing significantly to the elucidation of numerous biological processes. Complement protein C3, as well as other proteins of the C3/α-2M thioester protein family all possess an eponymous thioester bond that bridges the cysteine and glutamine side chains of the conserved sequence GCGEQ to form the internal β-cysteinyl-γ-glutamyl, fifteen-membered thiolactone ring, a distinctive post-translational modification of this class of proteins that makes this family theoretically amenable to site-specific labelling (Fig. 1). In almost all cases of activity-based labelling, a protein activity is targeted by an electrophilic warhead (Heal et al. 2011), however for the C3/α-2M family this situation is reversed, as the thioester bond is itself the warhead and can be targeted with an appropriately reactive nucleophile. Thioester proteins are present in high concentrations in blood plasma as the broad-spectrum protease inhibitor α-2M (2.0 mg ml^−1^) and as C3 (1.2 mg ml^−1^) and C4 (∼0.6 mg ml^−1^) of the complement pathway. C3 is a 190 kDa protein consisting of a 113 kDa α-chain, containing the thioester bond, and a 75 kDa β-chain linked by a single disulphide bond. In recent years, the crystal structures (Fredslund et al. 2006; Janssen et al. 2005, 2006) of intact complement C3 and its activation product C3b (Fig. 1) have revealed the two end points of the structural transition that takes place when the C3 molecule is activated and cleaved: upon going from C3 to C3b, the C3 domain containing the thioester (TED domain) undergoes a large rearrangement, while the domains surrounding the anaphylotoxin domain (ANA) also change orientation and expose surfaces for interaction with Factor B (fB), Factor I (fI) and its cofactors such as fH. Although it has not received the attention of C3, α-2M is an important serum protein that acts as a broad range protease inhibitor by physically trapping proteases and preventing them reaching substrate (Salvesen and Barrett 1980). α-2M has a ‘bait’ region with high substrate activity for a wide range of proteases. Analogous to the C3a or ANA domain in C3, cleavage of this bait region causes a dramatic conformational change. Related thioester containing proteins are represented across metazoans (Dodds and Law 1998) and include the thioester protein (Tep) of *Drosophila* (Baxter et al. 2007).

**Fig. 2:**
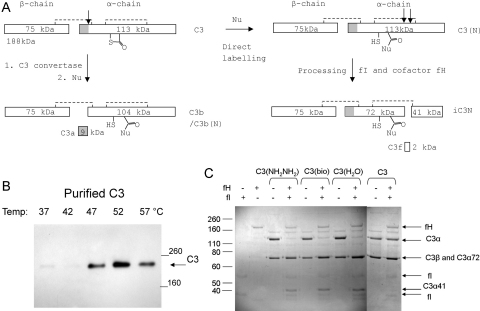
Chemical labelling of C3 thioester bond. (A) Schematic representation of C3 polypeptide structure and primary activation products. Interchain disulfide bonds are represented by dotted lines, C3a in grey and product sizes are indicated. Protease activation of C3 by C3 convertases followed by a rapid reaction of the activated thioester to surrounding nucleophilic groups to form C3b bound nucleophile C3b(N). The labelling of C3 by the direct reaction of nucleophile with the thioester without protease C3a cleavage gives C3(N). FI and cofactor fH process C3b to iC3b by protease cleavage. The C3(N) conjugate possesses a C3b like structure and can be processed in the same way (Law and Dodds, 1997). (B) Temperature dependence of biotin–PEG_4_–hydrazide labelling of purified C3. Purified C3 incubated with biotin–PEG_4_–hydrazide for 1 h at indicated temperatures. Samples analysed by 6% SDS-PAGE by Western blots of SA-HRP. (C) SDS-PAGE for comparison of C3(N) processing with fI and fH: C3(NH_2_NH_2_) C3(bio), C3(H_2_O) and C3. C3-conjugates were prepared by incubation with nucleophile at 52 °C for 1 h and dialysed in PBS before reaction with fI and fH for 1 h at rt. Processing of C3 detected by Coomassie staining.

The intact thioester proteins behave as typical protein thioesters with an estimated half-life of hydrolysis of the thioester of intact C3 (∼160 h) within the range of a peptide thioester (Davies and Sim 1981). Characteristically for thioesters they are much more susceptible to aminolysis than hydrolysis (Yang and Drueckhammer 2001) and this observation has been used in many studies to incorporate low molecular weight nucleophiles (Law and Dodds 1997). Furthermore, the slow hydrolysis of intact C3 that constitutes the ‘tick over’ event of the alternative pathway to form C3(H_2_O) shows that the thioester bond is in contact with solvent. C3(H_2_O) can form a convertase C3(H_2_O)Bb (Fig. 1) like its truncated relative C3b and is a substrate for fI, and although no structure has yet been reported for C3(H_2_O) it is assumed to behave similarly to C3b (Bexborn et al. 2008). Cleavage (in this case hydrolysis) of the thioester bond is enough to cause transition to a C3b-like structure. This pathway would therefore seem an ideal route to incorporate nucleophiles attached to a bioreporter (as suggested in Figs. 1 and 2A). The thioester bond is a very rare post-translational modification for an extracellular protein and reactive to nucleophiles so that it can be chemoselectively derivatized. Nevertheless chemical approaches to labelling and regulating complement have been largely neglected. When activated by limited proteolysis the thioester becomes extremely reactive with a very short serum lifetime, and will react rapidly with many nucleophiles (Sim and Law 1985; Sim et al. 1981). Conjugation of C3b to ovalbumin and other targets (Cretin et al. 2007; Villiers et al. 1999) has been achieved by adding trypsin to C3 in an excess of the conjugating agent to take advantage of this activation, although coupling efficiency is low. Radiolabelled methylamine has been incorporated into serum fractions of many organisms to screen for the presence of thioester proteins across metazoans (Dodds et al. 1998). Recently, an ingenious method has been used to label C3 efficiently with larger molecules, C3 is treated with methylamine, and as the thioester is aminolysed the cysteine side-chain becomes transiently available for derivatization. This promising approach has been used to conjugate C3 to harness its immunological properties (Mitchell et al. 2008). We have previously demonstrated, that purified C3 thioester can be directly reacted via the thioester bond with much larger biomolecules than were used before. However it was still difficult to measure the efficiency of the reaction, estimated by indirect methods such as ability to perform the autocleavage reaction (Cole et al. 2009). Attempts at pull-down directly from sera gave complex product mixtures from post-derivatization processing. We considered that it would be worthwhile to reinvestigate serum pull-down of full-length derivatized protein with the knowledge that high salt inhibits formation of C3(N)fHfI (Soames and Sim 1997). This approach would yield efficient labelling by pull-down of the intact, derivatized material, directly from serum.

Our aim is to develop a detection reagent that reacts with thioester proteins in serum, without need to purify the protein. An activity-based approach using a nucleophile attached to a reporter molecule would potentially be able to extract and detect the active thioester proteins present (Heal et al. 2011). This chemical approach would accelerate and simplify complement detection by obviating the need for expensive antibodies and dedicated machinery. More importantly this technique will allow us to directly quantify active complement C3 or C4 in serum. The pivotal role of complement in the immune response is reflected in the number of medical conditions correlated with abnormal complement activation or consumption (Morgan and Harris 2003; Nilsson et al. 2011). Thioester labelling would also be useful for screening and comparison of thioester proteins from a wide variety of metazoans, but most importantly for direct longitudinal studies of active C3, C4 levels in different diseases.

## Materials and methods

### Blood/plasma/serum preparation

Blood was drawn from healthy volunteers and either used immediately or the plasma/serum separated and stored at −80 °C. For serum samples the freshly drawn blood was left at room temperature for 30 min, cells and resulting clot were pelleted by centrifugation (2000 × *g*, 15 min) and the serum stored at −80 °C. Plasma was prepared by collecting freshly drawn blood (typically 15 ml) in centrifugation tubes (Corning, 15 ml) containing 1.8 mg sodium EDTA/ml blood (final EDTA concentration about 5 mM). Tubes were inverted carefully to mix the samples and left on ice (10 min) before centrifugation (2000 × *g*, 10 min). The plasma fraction was retained. Paired plasma samples (pre-operative, POD0 and 48–60 h post-operative, POD3) were obtained from patients enrolled in the Post-Operative Morbidity: Exercise (POM-E) Study [UKCRN ID 8165/ISRCTN 90019424]. Subjects in this observational study were recruited in accordance with Local Research Ethics Committee approval and underwent elective total hip or knee replacement at University College Hospital London. Following informed consent, freshly drawn blood samples were mixed with heparin (final concentration 50 iu/ml blood) and kept on ice before centrifugation (2000 × *g*, 10 min at 4 °C). The upper plasma fraction was retained and stored at −80 °C.

### Purification of C3, fH, fI

Human C3 was purified as previously described (Dodds 1993). Briefly, plasma (typically 7.5 ml) was precipitated with a half vol. of 15% PEG 3350 (Sigma) in Buffer A (20 mM tris, 50 mM EACA, 5 mM EDTA, 0.02% NaN_3_, 0.2 mM PMSF, pH 7.5) to give a final PEG concentration of 5%. Samples were kept on ice (0 °C, 30 min) with gentle agitation and the resulting precipitate was pelleted by centrifugation (9500 × *g*, 20 min, 4 °C). PEG precipitated plasma (PEG-plasma) was filtered, degassed and anion exchange chromatography was performed on an Äkta (GE, Uppsala) with a Q-Sepharose column (20 cm × 1.6 cm diam.), eluting with a NaCl gradient (10 CV, 5–50% Buffer B, where Buffer B is 1 M NaCl in Buffer A, flow 1 ml/min). Fractions containing C3, as assessed by SDS-PAGE analysis, were pooled and diluted with 1 vol of water before purification with higher resolution ion exchange chromatography using a monoQ column (MonoQ 10/100GL), equilibrated in 90% A, 10% B. Bound proteins were eluted with a NaCl gradient (20 ml, 10–30% buffer B, flow 1 ml/min). Purity of the C3 preparation was determined by SDS-PAGE.

fI and fH were prepared as described by (Sim et al. 1993) and were generously provided by Dr. Pietro Roversi (Pathology, Oxford University).

### Chemical functionalization of thioester bond

The thioester protein samples (blood/plasma/serum or purified C3) (200 μl) were diluted with PBS (200 μl), mixed with an equal volume of NaCl (2 M, 400 μl) and biotin–PEG_4_–hydrazide (Celares, GmbH, Berlin) (250 mM, 16 μl) giving a final NaCl concentration of 1 M and a probe concentration of 5 mM. The mixture was incubated at 37 °C, 42 °C, 47 °C, 52 °C or 57 °C for 1 h or 37 °C for 24 h. Un-reacted biotin–PEG_4_–hydrazide was removed by dialysis against Ca and Mg-free Dulbecco's PBS pH 7.4 (5 × 250 ml) at 4 °C using Slide-A-Lyser^®^ MWCO 10 k (Pierce). Labelled samples were stored at 4 °C until use. For the preparation of reference proteins: hydrolysed C3 (C3(H_2_O)) and hydrazine treated C3 (C3(NH_2_NH_2_)) were performed as above with the addition of either H_2_O or a hydrazine solution (250 mM, pH 7) for 1 h at 52 °C.

### Western blot

Thioester-containing proteins from blood/serum or plasma were labelled by incubation with biotinylated probes as described above. Samples were boiled for 7 min in 2 times SDS loading buffer with or without 5% β-mercaptoethanol, for reducing or non-reducing conditions respectively. Novex^®^ Sharp Pre-Stained Protein Standard (Invitrogen) was used as molecular weight standard. Following SDS-6% PAGE using the procedure of Laemmli (1970) the proteins were blotted onto nitrocellulose membrane (0.45 μm, Thermo Scientific). The membrane was blocked with 3% bovine serum albumin (BSA) in PBS for 1 h at rt and washed 3 times with PBST (PBS with 0.05% Tween 20). The membrane was incubated with SA-HRP (Pierce) 1:50,000 in 1% BSA in PBS (1 h, rt), washed 5 times with PBST, rinsed (PBS), developed using SuperSignalWestPico (Pierce), and detected by exposure to CL-Xposure™ film (ThermoScientific).

### Pull-down of biotinylated proteins from blood, serum or plasma

Sufficient slurry of Pierce High Capacity Streptavidin–Agarose resin (Pierce, IL) to give approximately 75 μl settled resin in a microtube (1.5 ml) was prepared for each sample. Resin was washed 3–5 times in PBS (400 μl) followed by centrifugation (500 × *g*, 1 min). Blood, plasma, serum or purified C3 derivatised with biotin–PEG_4_–hydrazide (500 μl), was mixed with 10% SDS (50 μl) to give a final SDS concentration of 0.9%. The mix was added to the washed SA resin and incubated at rt with end-over rotation for 2 h. The slurry was centrifuged (500 × *g*, 1 min.), supernatant removed and the resin re-suspended in PBS (200 μl). The slurry was transferred to a spin column (spin cups-cellulose acetate filter, Pierce) washed 5 times with PBS (400 μl) each followed by centrifugation (1000 × *g*, 3 min) and removing the flow through. The resin was finally re-suspended in 2% SDS (400 μl), transferred to a microtube (1.5 ml), allowed to settle and the supernatant decanted. Captured proteins were eluted from the resin in Laemmli buffer (40–50 μl) (62.5 mM Tris, 25% glycerol, 2% SDS, 0.01% Bromophenol Blue) by boiling for 7 min. Immediately after boiling the slurry was centrifuged (1500 × *g*, 1 min) and the supernatant analysed by SDS-6% PAGE or Nu-PAGE 4–12% gradient gel (Invitrogen) for quantification. Gel staining was performed using Coomassie blue R-250 or for quantification Lumetein™ protein gel stain (Biotium). Band intensities were measured on a STORM 860 (Amersham Biosciences) and quantified using ImageQuantile TL (Amersham Biosciences). A paired-sample *t* test was performed on patient samples. Patient C3 concentrations were estimated from C3 reference added as an internal gel standard and the C3 calibration standard curve.

### Quantification of C3 in plasma

Paired patient plasma samples (POD0 and POD3) and C3 were labelled with biotin–PEG_4_–hydrazide as described above. The C3(bio) concentration was determined from *A*_280_ and a 2× dilution series of C3(bio) in PBS was prepared starting from 1.25 μM (500 μl, 117.5 μg). 500 μl of the prepared C3(bio) dilutions and 500 μl of biotin–PEG_4_–hydrazide labelled plasma were subjected to SA-pull down (as described above) and analysed on Nu-PAGE 4–12% gradient gel (Invitrogen). The gels were stained in Lumetein™ protein gel stain (Biotium). Band intensities of pull-down C3 proteins were measured on a STORM 860 (Amersham Biosciences) and quantified using ImageQuantile TL (Amersham Biosciences). A paired sample *t*-test was performed on band intensities for the patient samples. The difference between POD0 and POD3 was compared using the *t*-test in excel (one-tailed, type 1 paired). Patient C3 concentrations were estimated from a C3 reference added as an internal gel standard and the C3(bio) calibration standard curve. Each paired sample was normalised with respect to the POD0 sample and the average change in POD3 was calculated and displayed with standard deviation.

### Peptide mass fingerprinting

Protein bands were excised from the Coomassie stained SDS gel and cut in small pieces. In-gel tryptic digestion was carried out as described (Shevchenko et al. 2006). Briefly, the gel pieces were washed (MeCN and NH_4_HCO_3_), reduced with DTT (10 mM in NH_4_HCO_3_, 56 °C, 1 h), alkylated by iodoacetamide (55 mM in NH_4_HCO_3_, rt, 30 min) and treated with trypsin (5 ng/μl in NH_4_HCO_3_) 37 °C. Tryptic digests were desalted and concentrated using a micro C_18_ column (ZipTip, Millipore, MA). Peptides were eluted from the C_18_ column with matrix (saturated solution of α-cyano-4-hydroxy-cinnamic acid in 50% H_2_O, 50% MeCN, 0.1% TFA, 2–5 μl) and directly deposited onto a steel target for analysis by MALDI-TOF MS (microflex LRF, Bruker Daltonics, MA). Spectra were acquired in reflectron mode between *m*/*z* 500 and 4880. Raw spectra were processed using BioTools (Bruker Daltonics, MA) and centred peptide masses exported to the MASCOT search engine (Matrix Science, UK). Data were searched against the SwissProt database and limited to human taxonomy, allowing CAM-cysteine (carbamidomethylcysteine) as a fixed and oxidised methionine as a potential variable modification. A peptide mass-tolerance of 150 ppm was allowed.

### Factor I processing of C3-conjugates

Factor I processing of C3-conjugates was carried out as described previously (Tsiftsoglou et al. 2005, 2006). Briefly, C3 protein or conjugate; C3(H_2_O), C3(NH_2_NH_2_) or C3(biotin–PEG_4_–hydrazide) (2.75 μg), was added to a mix of fI (0.5 μg) and fH (0.5 μg) in reaction buffer (10 mM potassium phosphate, 0.5 mM EDTA, pH 6.5) with a final reaction volume of 50 μl. The reactions were incubated at 37 °C, with shaking (1 h) and quenched (−80 °C). 5× SDS loading buffer (10 μl; 250 mM TrisHCL, 10% SDS, 30% glycerol, bromophenol blue) containing DTT (40 mM) was added and the samples boiled prior to analysis by SDS-8.5% PAGE. Gel bands were visualised with Coomassie blue R-250 stain.

## Results

### C3 thioester labelling and reaction temperature optimisation

The overall aim was to detect whole, C3/α-2M thioester proteins on an SDS gel from a serum pull-down by targeting the chemical reactivity of the thioester bond with nucleophiles attached to a reporter group (Biotin). This approach would use the same route as causes the formation of C3(H_2_O) in the alternative pathway (Figs. 1 and 2A). The chemoselective derivatization of the thioester bond risked extensive processing of C3(N) by proteases, because when the thioester bond reacts with the nucleophile the C3-conjugate formed adopts a C3b-like structure that has binding sites available for the serine proteases fI and fB (Nishida et al. 2006). The products of C3(N) proteolysis would complicate the interpretation of an SDS gel. Therefore, incubations with nucleophiles in serum were performed at high NaCl concentration that acts to suppress formation of C3(N)fB (Maeda and Nagasawa 1990) or C3(N)fHfI (Soames and Sim 1997). However, overnight incubation of serum with nucleophiles at room temperature and high NaCl resulted in poor labelling and some degradation of C3 (data not shown). Therefore the reaction was heated to increase the rate of reaction. A series of reaction temperatures (all below 64 °C to prevent denaturation (Sim and Sim 1981)) were chosen and monitored for thioester bond reaction after 1 h. Purified C3 was incubated at high NaCl concentration in presence of biotin–PEG_4_–hydrazide (5 mM final conc.) for 1 h at different temperatures. The sample was then exhaustively dialysed to remove any excess biotin–PEG_4_–hydrazide that could coat the SA-resin and cause poor pull-down. Purified C3 protein incubated with biotin–PEG_4_–hydrazide was detected by Western blot using SA-HRP (Fig. 2B). Only traces of labelled C3 were observed at 37 °C and 42 °C. At 47 °C and 52 °C a clearly labelled single band of C3 corresponding to full size 188 kDa protein was observed, optimal at 52 °C (Fig. 2B).

The experiment was repeated but with PEG-plasma (excluding only high molecular weight proteins) instead of purified C3 (supporting material). The same temperature dependence was observed with PEG-plasma as for purified C3 (Fig. 2A). While the reaction of biotin–PEG_4_–hydrazide with the thioester bond of C3 was barely detectable at 37 °C, labelling was optimal at 52 °C, 1 h. No other C3 products were detected by Western blot in the PEG-plasma so the conditions of high NaCl, 52 °C and 1 h incubation time were subsequently used throughout these studies.

### Labelled C3-conjugates are processed by factor I.

In serum, C3b is rapidly processed by fI preventing further C3 convertase (C3bBb) formation. Thioester bond-functionalized C3 (C3(N)) such as hydrolysed C3, C3(H_2_O) and C3(biotin–PEG_4_–hydrazide) possess C3b-like structure (Nishida et al. 2006; Parkes et al. 1981). C3(N) displays binding sites for fH cofactor that allow further processing by fI initially to an iC3b-like molecule (iC3N in Fig. 2A). To test if the C3-conjugates C3(N) produced under these labelling conditions were behaving similarly to C3b and were not denatured, the C3(N) were tested for fI cleavage. C3 was reacted with biotin–PEG_4_–hydrazide, hydrazine or hydrolysed at 52 °C for 1 h, in high NaCl buffer and dialysed exhaustively before cleavage by fI in presence of fH (37 °C, 1 h). The fragmentation pattern was analysed by SDS-PAGE. In the absence of fI and fH no cleavage products were observed and only intact α- and β-chain were detected (Fig. 2C). In the presence of fI and fH, the α-chain was almost completely processed and the 41 kDa fragment was observed for all the C3(N) samples. The other fragment formed, 72 kDa, co-ran with the β-chain and so was not seen as a distinct product. As expected, the reference C3 protein (which was not subjected to labelling conditions) showed no susceptibility to fI cleavage. Derivatized C3, C3(NH_2_NH_2_) and C3(bio) was processed by fI. Under the same labelling conditions in the absence of nucleophile, complete hydrolysis occurred, forming C3(H_2_O), as judged by susceptibility to fI.

### Comparison of chemical labelling of thioester proteins in blood, sera, plasma or PEG-plasma

**Fig. 3:**
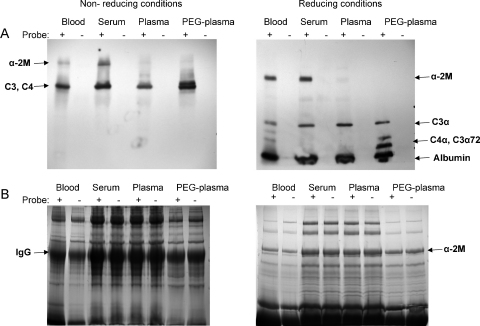
C3/α-2M thioester protein labelling in blood, plasma, serum and PEG-plasma. Blood, plasma, serum and PEG-plasma incubated with (+) or without (−) biotin–PEG_4_–hydrazide for 1 h at 52 °C. (A) Biotinylated proteins were detected by SA-HRP Western blot. Non-reducing (left panel) and reducing (right panel) conditions. (B) SDS-PAGE showing total protein content detected by Coomassie staining.

We were concerned that the different preparations of human blood used could affect the assay. Therefore the optimized reaction conditions were used for comparison of the reaction of the thioester bond containing proteins in human blood, serum, plasma and PEG-plasma. Biotin–PEG_4_–hydrazide was incubated with blood, serum, plasma or PEG-plasma in high NaCl buffer for 1 h at 52 °C. The reaction mixtures were analysed by SDS-PAGE and Western blot, labelled proteins were detected by SA-HRP (Fig. 3). The Coomassie stained gels reveal a large amount of total protein in all samples (Fig. 3B), the major-band on the non-reducing gel, is IgG (∼150–160 kDa). On the reduced gel, the major band, present in all the samples, is the α-2M monomer, this is the most abundant serum protein above 100 kDa on a reducing gel (∼180 kDa). The Western blot suggested that at least two proteins were specifically labelled, and additionally some weaker bands of high molecular weight were also detected (Fig. 3A). Under non-reducing conditions (left panel) a protein corresponding to a dimer of α-2M can be seen clearly labelled for the blood and serum samples. This α-2M dimer of around 360 kDa is too big to resolve on this gel. A protein corresponding to C3 in size is strongly labelled by biotin–PEG_4_–hydrazide in all the samples. However C3, C3b, C4, C4b and iC3b all run close together on a non-reducing gel so it is not possible to distinguish between them or to know whether they were processed further from residual protease, which appears to be the case for the PEG-plasma sample. Under reducing condition (right panel) the pattern clearly shows presence of α-2M monomer and C3 α-chain (the C3 α-chain containing the thioester bond). This Western blot had poor resolution at lower molecular weights (Fig. 3A, right panel), a band less than 80 kDa was labelled that could potentially be identified as the C3α71 fragment. With improved separation the labelled band was shown to travel more than the 60 kDa-marker and could be either albumin (supporting material) or possibly C3bα62 (Fig. 2A) the C3b α-chain fragment. The high-salt largely suppressed processing under the conditions used, however there are bands that perhaps could be the C3α72 fragment indicating residual protease activity, especially on the blood and PEG-plasma samples. The pull-down from serum (Fig. 3A) showed good labelling of both C3 and α-2M proteins. However, the plasma specimens showed little labelling of α-2M, the presence of anticoagulants may interfere with labelling.

### C3 could be pulled-down from blood/plasma/serum/PEG plasma

**Fig. 4:**
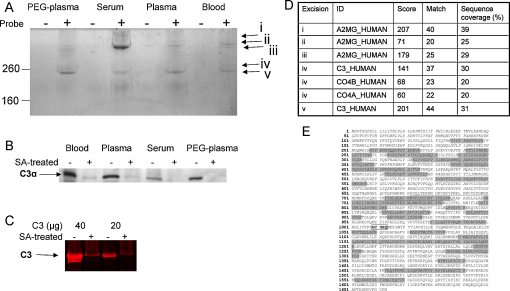
Biotinylated protein pull-down. (A) Blood, plasma, serum and PEG-plasma were incubated with (+) or without (−) biotin–PEG_4_–hydrazide probe for 1 h at 52 °C. Biotinylated proteins were captured by SA-resin, eluted by boiling the resin in SDS loading buffer and visualised by 6% SDS-PAGE and Coomassie staining. (B) Western blot of biotin–PEG_4_–hydrazide treated blood, plasma, serum and PEG-plasma pre (−) and post (+) SA capture. Biotinylated proteins detected by SA-HRP. (C) Total C3 protein in reaction mix pre (−) and post (+) SA capture. Two known amounts of C3(bio) were treated with SA-resin and the *non*-captured remaining protein detected by SDS-PAGE, with lumeitin stain. (D) Table of identified peptides from pull-down experiment (A). Gel bands i–v were excised, and mass fingerprint established by tryptic digest and MALDI-TOF MS, searched against the Swissprot database by the Mascot search engine. All identified proteins (with scores greater than 56 and i.e., significant (*p* < 0.05)) are listed with ID, score, number of matched peptides and % sequence coverage. (E) Sequence coverage of the identified C3 protein from excision v. β-chain shown in italics, identified peptides highlighted in grey, the GCGEQ (the internal thioester) is boxed, and the Ana (C3a) domain underlined (cleaved during C3 activation by C3 convertase).

**Scheme 1:**
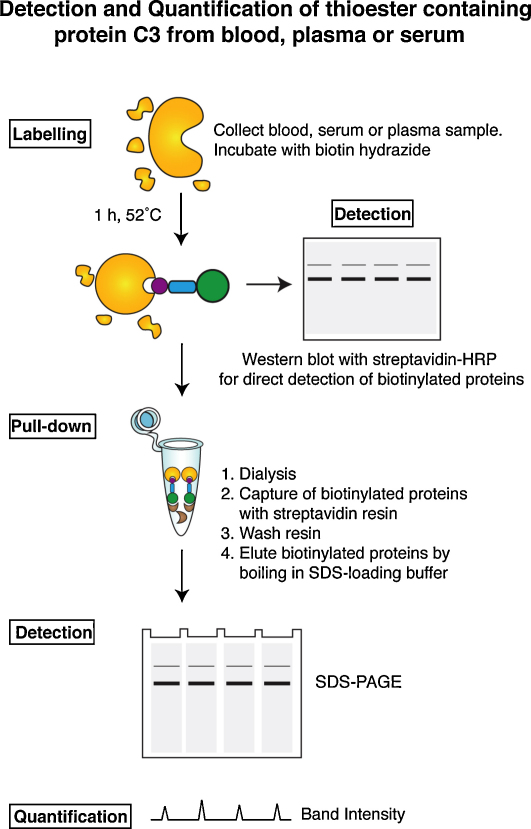
Procedure for labelling, pull-down and quantification of thioester containing proteins from blood, serum or plasma.

A streptavidin pull-down assay was developed for biotin–PEG_4_–hydrazide reacted samples to identify the labelled proteins in blood, plasma and serum, as well as develop a direct method for quantification (Scheme 1). Biotinylated proteins were recovered by SA-resin, eluted with SDS-loading buffer and visualized by SDS-PAGE and Coomassie stain (Fig. 4A). The efficiency of the pull-down assay was tested by comparison of the samples by Western blot (SA-HRP) before and after SA-resin pull-down (Fig. 4B). By Western blot almost all the biotinylated C3 was pulled-down from blood and plasma and no residual labelled proteins were detected after pull-down. Total protein in the C3 lane for two C3(bio) samples before and after SA-pull-down were compared by inspection of the stained C3 gel band on SDS-PAGE (Fig. 4C). Only small quantities of protein were observed co-running with the C3 band after SA-pull-down that may correspond to C3(H_2_O), unlabelled C3 or unrecovered C3. This result indicated that hydrolysis was not a major competing reaction in the presence of a good nucleophile as would be expected for a thioester (Yang and Drueckhammer 2001). High molecular weight proteins were pulled down in all the tested specimens: blood, plasma, serum and PEG-plasma (Fig. 4A). In the absence of biotin–PEG_4_–hydrazide there was no non-specific binding. In addition, a low molecular weight protein was detected (visualized < 60 kDa-marker) identified as albumin (supporting figure). Five bands (i–v) (Fig. 4A) were cut from the SDS gel, and the mass fingerprint of each protein recorded by tryptic digest and MALDI-TOF MS. The three known thioester containing proteins α-2M, C4 and C3 were identified as significant matches (Fig. 4D). While C3 was detected readily in blood, plasma, serum and PEG-plasma (in excision v) α-2M (found in three different forms, excision i-iii) was preferably detected in serum. C4 was identified in a weakly stained gel band (excision iv) just above the major C3 band. C3 was verified to very high scores and the identified peptides clearly cover both C3 α-and β-chain (Fig. 4E). The sequence coverage of the α-2M and C4 proteins is given in the supporting material.

### Comparison of reactivity of thioester proteins with biotin–PEG_4_–hydrazide vs biotin–PEG_3_–aminoxy

**Fig. 5:**
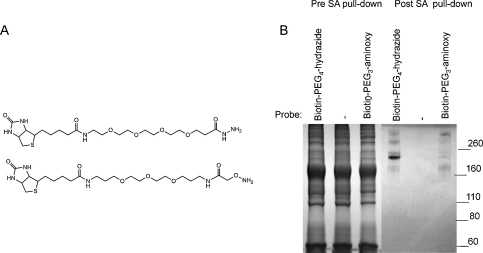
Comparison of thioester protein functionalization for biotin–PEG_4_–hydrazide and biotin–PEG_3_–aminoxy. (A) Chemical structures of biotin–PEG_4_–hydrazide (top) and biotin–PEG_3_–aminoxy (bottom). (B) PEG-plasma was incubated with biotin–PEG_4_–hydrazide, biotin–PEG_3_–aminoxy. Biotinylated proteins were captured with SA-resin, eluted by boiling in SDS loading buffer and visualised by 6% SDS-PAGE and Coomassie staining. Total protein present in reaction mix pre-SA capture (left 3 lanes) is shown beside the pull-down proteins (right three lanes).

Although biotin–PEG_4_–hydrazide reacted well we considered that using a stronger alpha effect nucleophile could optimize labelling further and perhaps show some useful discrimination of labelling between the thioester proteins. Biotin–PEG_3_–aminoxy (Fig. 5A) and biotin–PEG_4_–hydrazide were incubated with PEG-plasma and subjected to the SA-pull-down assay essentially as described before (temperature 57 °C, final probe concentration 1.8 mM) (Scheme 1). Total protein before SA-pull-down and the captured proteins were analysed by SDS-PAGE and visualized by Coomassie staining (Fig. 5B). Surprisingly, although more nucleophilic, the biotin–PEG_3_–aminoxy probe showed little reactivity towards the C3 thioester, but it reacted with α-2M. This suggests that the biotin–PEG_3_–aminoxy probe may be further developed as an α-2M selective probe. Differential reactivity of activated C3, C4A and C4B for amine or oxygen nucleophiles has been observed previously, so the selectivity observed here may be under the same sequence control (Law and Dodds 1997).

### Measurement of active C3 in patient serum samples pre- and post-surgery

**Fig. 6:**
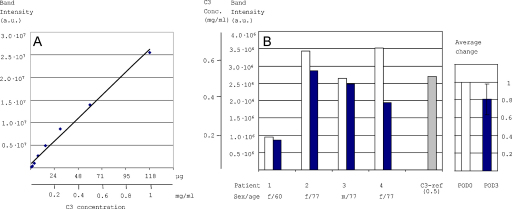
Quantitative detection of C3 in patient serum samples. (A) A linear correlation verified between C3 concentration and detected gel band intensity after SA pull-down assay and SDS-PAGE. Varying concentrations (2× dilution series) of purified C3(bio) were subjected to the SA-pull-down assay and the captured protein analysed by SDS-PAGE and Lumetein staining. Quantified gel band intensities plotted against C3 concentration and the linear trend line displayed. (B) Plasma samples from four surgical patients (1–4) undergoing elective joint replacement were tested for C3 levels before operation (POD0, unfilled bar) and 48–60 h after operation (POD3, filled bar). Four paired patient plasma samples (POD0 and POD3) were incubated with biotin–PEG_4_–hydrazide and labelled proteins were pulled-down and detected as in A. The quantified C3 gel band intensities (estimated C3 concentrations) were plotted against patient and condition (left panel). The difference seen between POD0 and POD3 was statistically verified in a paired sample *t*-test to *p* = 0.09. The average change shown as normalised values of the mean POD0 and POD3 (right panel).

To validate the C3 pull-down assay for quantitative measurements, we defined the linearity range within the applicable concentration range. A 2-fold dilution series of a known concentration of C3(bio) was prepared and each sample subjected to SA pull-down assay. The captured proteins were detected on gel with Lumitein protein gel stain (which allows a linear range of detection for at least 3 orders of magnitude) and the band intensities were quantified (Fig. 6A). The dilution series clearly show a linear response with good fit. Complement activation is part of the systemic inflammatory response. We measured C3 levels pre and post-operative as a system for testing the pull-down quantification assay. Plasma samples were taken from four patients pre-surgery (POD0) and 48–60 h post-surgery (POD3). The samples were incubated with biotin–PEG_4_–hydrazide, dialysed and subjected to the SA pull-down assay (Scheme 1). The gel band intensities of the recovered C3 proteins were quantified and the paired pre- (POD0) and post-operative samples (POD3) were plotted side by side (Fig. 6B). As expected the inter-patient variation of C3 levels (POD0) was large. Moreover, all patients showed a lower active C3 concentration post operation (POD3) compared to their pre-surgery values. The average C3 value decreased by 20% (Fig. 6B, right panel). The decrease in concentration of C3 between POD0 and POD3 was statistically verified to the 10% level in a paired sample *t*-test. These results indicate an initial decrease in the level of active C3 as part of the post-operative inflammatory response. This preliminary study shows potential for the use of chemical probes for selective labelling of active C3 for quantification.

## Discussion

The chemistry of peptide thioesters is increasingly familiar because of their use as intermediates for chemical protein synthesis (Dawson and Kent 2000). Peptide thioesters are generally quite stable to hydrolysis at physiological pH and favour aminolysis over hydrolysis (Yang and Drueckhammer 2001). The C3/α-2M thioester proteins all exist in plasma in an unactivated ‘native’ form, that resemble, in their chemical reactivity to nucleophiles, peptide thioesters (Davies and Sim 1981). This resemblance was what initially suggested the possibility of covalently attaching large biomolecules to C3. In contrast, the exceptional reactivity of the short-lived, activated form of the C3/α-2M thioester proteins after limited proteolysis probably reflects the increase in bond strain of the helix spanning thioester during transition from a 3_10_ helix in the thioester domain of native C3 to an α-helix (Fredslund et al. 2006).

We have demonstrated that a single chemoselective chemical reaction can be used to select and label a small group of immunoproteins from all the other proteins present in human serum. This activity based assay can efficiently pull-down all the biotinylated proteins in the sample and we have demonstrated that biotinylation of C3 was quantitative using optimised labelling conditions. The use of high salt to inhibit processing and higher temperature to shorten labelling time still gave the correctly folded biotinylated products as demonstrated by fH and fI processing. The presence of high salt suppresses post-processing of the conjugated thioester proteins so they can be detected whole (some degradation was observed on incubation in PEG-plasma (Fig. 3)). Furthermore, the demonstration of a consistent drop in C3 concentration in human samples after elective surgery indicates that this pull-down assay could potentially form the basis of a quantitative C3 assay. The importance of complement to the immune response is reflected in the number of medical conditions correlated with abnormal complement levels. For example, elevated serum complement C3 confers risk for coronary heart disease and type 2 diabetes (Onat et al. 2005). The method described herein, using SDS-PAGE, although useful for demonstration and proof of principle would not be suitable for running high numbers of clinical samples. However, it should be adaptable to an ELISA assay, for example capturing labelled C3 on plates coated with anti C3-Mab and quantifying with avidin–HRP or alternatively where the capture step is based on streptavidin binding and the development step on antibodies raised against a range of C3/C4/α-2M forms. With the demonstration of efficient ligation of labels to the thioester proteins other potential applications can be envisaged. C3 (as C3b and C3d) is an excellent natural, non-toxic adjuvant (Cretin et al. 2007; Villiers et al. 1999). Many efforts have been made to selectively incorporate antigen to C3 or its processed derivatives. Using direct conjugation at the thioester position under the optimised conditions established it should now be possible to attach antigens covalently either directly or using bi-functional linkers. An advantage of this approach is that several copies of C3 can be attached in a chemically defined way to the antigen, allowing titration of the optimal adjuvant response. Additionally, ligation to non-protein antigens such as carbohydrates, important targets of cancer, viral and HIV vaccine development, can be explored (Astronomo and Burton 2010). A similar approach of attaching imaging agents to C3 will help to further elucidate the complement pathway and observe some of the cellular responses to C3.

In conclusion, we have developed a method to target thioester-containing proteins and were able to capture and isolate full-length C3, C4 and α-2M from plasma. We anticipate that this method could be useful for screening and comparison of thioester proteins from a wide variety of metazoans and most importantly to measure the amounts of active C3, in different disease states. The reactivity of the hydrazide moiety and other nucleophiles to thioesters may also prove to be useful in the development of complement specific inhibitors.
