# Supplementary material for: Chemical labelling of active serum thioester proteins for quantification
Source: Immunobiology. 2012 Feb;217-884(2):256–64. doi: 10.1016/j.imbio.2011.07.021 (PMC3274692; doi:10.1016/j.imbio.2011.07.021)
Supplement: Supplementary file 1 [file mmc1.pdf]

Supporting information

Chemical labelling of active serum thioester proteins for quantification.

Lotta Holm, Gareth L. Ackland, Mark R. Edwards, Ross A. Breckenridge, Robert B. Sim and John Offer.

Supporting Figure 1:

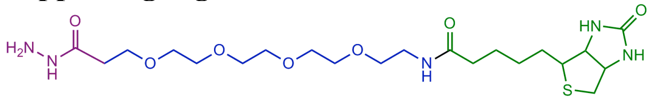

Chemical structure of thioester reactive probe: biotin-dPEG$_4$-hydrazide (Celares)

Supporting Figure 2:

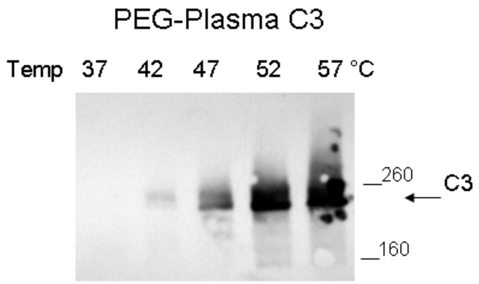

Supporting Figure 2:

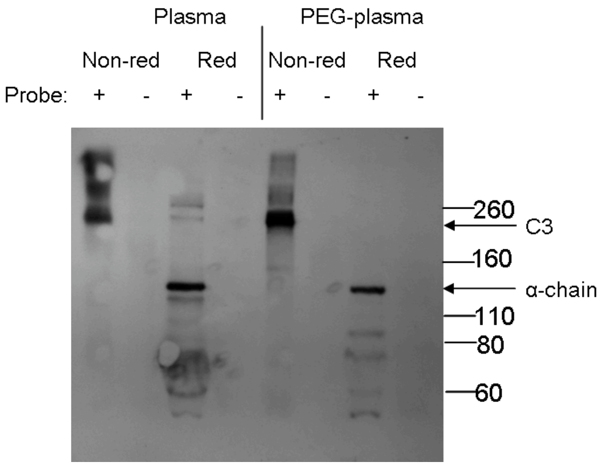

Supporting Material and Methods:

Peptide Mass Fingerprinting

MALDI-MS raw spectra for mass fingerprinting were processed using BioTools (Bruker Daltonics, MA). Data were processed using the smoothing algorithm SavitzkyGolay, baseline subtractions by the TopHat algorithm and peaks were centred at 80% peak high

Excision iv)
It could not be conclusively established which of the particular C4 isoforms (C4A, C4B or both) were present in this donor due to their extremely high sequence identity.
There is an extremely high sequence similarity between the two C4 isoforms (CO4A_HUMAN vs CO4B_HUMAN) in humans. A blast search was preformed to find the positions of isoform unique amino acids and further compared peptide containing a unique site were identified in the MALDI-TOF experiment. There were no peptides from CO4A_HUMAN found in a sequence unique area. There was only one unique peptide from CO4B_HUMAN found but would need LC-MS-MS to satisfactorily characterise and identify peptides.

Supporting Figure 3:
Peptide coverage of identified peptides for excision i-iv, vi.

Excision i)

Match to: **A2MG_HUMAN** Score: **207** Expect: **4e-17**
Alpha-2-macroglobulin OS=Homo sapiens GN=A2M PE=1 SV=3
Nominal mass (M_r): **164613**; Calculated pI value: **6.03**
NCBI BLAST search of A2MG_HUMAN against nr
Unformatted sequence string for pasting into other applications
Taxonomy: Homo sapiens
Fixed modifications: Carbamidomethyl (C)
Variable modifications: Oxidation (M)
Cleavage by Trypsin: cuts C-term side of KR unless next residue is P
Number of mass values searched: **100**
Number of mass values matched: **40**
Sequence Coverage: **38%**
Matched peptides shown in **Bold Red**

1 MGKNKLLHPS LVLLLLVLLP TDASVSGKPQ YMVLVPSLLH TETTEKGCVL
51 LSYLNETVTV SASLESVRGN R**SLFTDLEAE NDVLHCVAFA VPKSSSNEEV**
101 **MFLTVQVKGP TQEFK**KRTTV MVKNEDSLVF VQTDKSIYKP GQTVKFR**VVS**
151 **MDENFHPLNE LIPLVYIQDP K**GNR**IAQWQS FQLEGGLKQF SFPLSSEPFQ**
201 **GSYK**VVVQKK **SGGRTEHPFT VEEFVLPK**FE VQVTVPKIIT ILEEEMNVSV
251 CGLYTYGKPV PGHVTVSICR KYSDASDCHG EDSQAFCEK**F SGQLNSHGCF**
301 **YQQVK**TKVFQ LKRKEYEMK**L HTEAQIQEEG TVVELTGR**QS SEITRTITKL
351 SFVKVDSHFR QGIPFFGQVR LVDGKGVPIP NKVIFIRGNE ANYYSNATTD
401 EHGLVQFSIN TTNVMGTSLT VRVNYK**DRSP CYGYQWVSEE HEEAHHTAYL**
451 **VFSPSK**SFVH LEPMSHELPC GHTQTVQAHY ILNGGTLLGL K**KLSFYYLIM**
501 **AK**GGIVRTGT HGLLVKQEDM KGHFSISIPV KSDIAPVAR**L LIYAVLPTGD**
551 **VIGDSAKYDV ENCLANKVDL SFSPSQSLPA SHAHLR**VTAA PQSVCALRAV
601 DQSVLLMKPD AELSASSVYN LLPEKDLTGF PGPLNDQDNE DCINR**HNVYI**
651 **NGITYTPVSS TNEKDMYSFL EDMGLK**AFTN SKIRKPKMCP QLQQYEMHGP
701 EGLRVGFYES DVMGRGHARL VHVEEPHTET VRKYFPETWI WDLVVVNSAG

751 VAEVGVTVPD TITEWK**AGAF CLSEDAGLGI SSTASLRAFQ PFFVELTMPY**

801 **SVIR**GEAFTL KATVLNYLPK CIR**VSVQLEA SPAFLAVPVE K**EQAPHCICA

851 NGRQTVSWAV TPKSLGNVNF TVSAEALESQ ELCGTEVPSV PEHGRK**DTVI**

901 **KPLLVEPEGL EK**ETTFNSLL CPSGGEVSEE LSLKLPPNVV EESARASVSV

951 LGDILGSAMQ NTQNLLQMPY GCGEQNMVLF APNIYVLDYL NETQQLTPEI

1001 K**SKAIGYLNT GYQR**QLNYK**H YDGSYSTFGE R**YGR**NQGNTW LTAFVLK**TFA

1051 QARAYIFIDE AHITQALIWL SQRQKDNGCF RSSGSLLNNA IKGGVEDEVT

1101 LSAYITIALL EIPLTVTHPV VR**NALFCLES AWK**TAQEGDH GSHVYTK**ALL**

1151 **AYAFALAGNQ DKR**KEVLKSL NEEAVKK**DNS VHWERPQKPK** APVGHFYEPQ

1201 APSAEVEMTS YVLLAYLTAQ PAPTSEDLTS ATNIVKWITK **QQNAQGGFSS**

1251 **TQDTVVALHA LSK**YGAATFT R**TGKAAQVTI QSSGTFSSK**F QVDNNNRLLL

1301 QQVSLPELPG EYSMKVTGEG CVYLQTSLK**Y NILPEKEEFP FALGVQTLPQ**

1351 **TCDEPKAHTS FQISLSVSYT GSRSASNMAI VDVKMVSGFI PLKPTVK**MLE

1401 RSNHVSRTEV SSNHVLIYLD K**VSNQTLSLF FTVLQDVPVR** DLKPAIVK**VY**

1451 **DYYETDEFAI AEYNAPCSK**D LGNA

Excision ii)

Match to: **A2MG_HUMAN** Score: **71** Expect: **0.0016**

Alpha-2-macroglobulin OS=Homo sapiens GN=A2M PE=1 SV=3

Nominal mass (M_r): **164613**; Calculated pI value: **6.03**

NCBI BLAST search of A2MG_HUMAN against nr

Unformatted sequence string for pasting into other applications

Taxonomy: Homo sapiens

Fixed modifications: Carbamidomethyl (C)

Variable modifications: Oxidation (M)

Cleavage by Trypsin: cuts C-term side of KR unless next residue is P

Number of mass values searched: **100**

Number of mass values matched: **20**

Sequence Coverage: **24%**

Matched peptides shown in **Bold Red**

1 MGKNKLLHPS LVLLLLVLLP TDASVSGKPQ YMVLVPSLLH TETTEKGCVL

51 LSYLNETVTV SASLESVRGN R**SLFTDLEAE NDVLHCVAFA VPK**SSSNEEV

101 MFLTVQVKGP TQEFKKRTTV MVKNEDSLVF VQTDKSIYKP GQTVKFR**VVS**

151 **MDENFHPLNE LIPLVYIQDP K**GNRIAQWQS FQLEGGLKQF SFPLSSEPFQ

201 GSYKVVVQKK **SGGRTEHPFT VEEFVLPK**FE VQVTVPKIIT ILEEEMNVSV

251 CGLYTYGKPV PGHVTVSICR KYSDASDCHG EDSQAFCEK**F SGQLNSHGCF**

301 **YQQVK**TKVFQ LKRKEYEMK**L HTEAQIQEEG TVVELTGR**QS SEITRTITKL

351 SFVKVDSHFR QGIPFFGQVR LVDGKGVPIP NKVIFIRGNE ANYYSNATTD

401 EHGLVQFSIN TTNVMGTSLT VRVNYKDR**SP CYGYQWVSEE HEEAHHTAYL**

451 **VFSPSK**SFVH LEPMSHELPC GHTQTVQAHY ILNGGTLLGL KKLSFYYLIM

501 AKGGIVRTGT HGLLVKQEDM KGHFSISIPV KSDIAPVARL LIYAVLPTGD

551 VIGDSAK**YDV ENCLANKVDL SFSPSQSLPA SHAHLR**VTAA PQSVCALRAV

601 DQSVLLMKPD AELSASSVYN LLPEKDLTGF PGPLNDQDNE DCINRHNVYI

651 NGITYTPVSS TNEKDMYSFL EDMGLKAFTN SKIRKPK**MCP QLQQYEMHGP**

701 **EGLR**VGFYES DVMGRGHARL VHVEEPHTET VRKYFPETWI WDLVVVNSAG

751 VAEVGVTVPD TITEWK**AGAF CLSEDAGLGI SSTASLR**AFQ PFFVELTMPY

801 SVIRGEAFTL KATVLNYLPK CIR**VSVQLEA SPAFLAVPVE K**EQAPHCICA

851 NGRQTVSWAV TPKSLGNVNF TVSAEALESQ ELCGTEVPSV PEHGRKDTVI

901 KPLLVEPEGL EKETTFNSLL CPSGGEVSEE LSLKLPPNVV EESARASVSV

951 LGDILGSAMQ NTQNLLQMPY GCGEQNMVLF APNIYVLDYL NETQQLTPEI

1001 KSKAIGYLNT GYQRQLNYKH YDGSYSTFGE RYGRNQGNTW LTAFVLKTFA

1051 QARAYIFIDE AHITQALIWL SQRQKDNGCF RSSGSLLNNA IKGGVEDEVT

1101 LSAYITIALL EIPLTVTHPV VRNALFCLES AWKTAQEGDH GSHVYTKALL

1151 AYAFALAGNQ DKRKEVLKSL NEEAVKKDNS VHWERPQKPK APVGHFYEPQ

1201 APSAEVEMTS YVLLAYLTAQ PAPTSEDLTS ATNIVKWITK **QQNAQGGFSS**

1251 **TQDTVVALHA LSK**YGAATFT R**TGKAAQVTI QSSGTFSSK**F QVDNNNR**LLL**

1301 **QQVSLPELPG EYSMK**VTGEG CVYLQTSLK**Y NILPEKEEFP FALGVQTLPQ**

1351 **TCDEPK**AHTS FQISLSVSYT GSR**SASNMAI VDVKMVSGFI PLKPTVK**MLE

1401 RSNHVSRTEV SSNHVLIYLD K**VSNQTLSLF FTVLQDVPVR** DLKPAIVK**VY**

1451 **DYYETDEFAI AEYNAPCSK**D LGNA

Excision iii)

Match to: **A2MG_HUMAN** Score: **179** Expect: **2.5e-14**
Alpha-2-macroglobulin OS=Homo sapiens GN=A2M PE=1 SV=3
Nominal mass (M_r): **164613**; Calculated pI value: **6.03**
NCBI BLAST search of A2MG_HUMAN against nr
Unformatted sequence string for pasting into other applications
Taxonomy: Homo sapiens
Fixed modifications: Carbamidomethyl (C)
Variable modifications: Oxidation (M)
Cleavage by Trypsin: cuts C-term side of KR unless next residue is P
Number of mass values searched: **53**
Number of mass values matched: **25**
Sequence Coverage: **29%**

Matched peptides shown in **Bold Red**

1 MGKNKLLHPS LVLLLLVLLP TDASVSGKPQ YMVLVPSLLH TETTEKGCVL
51 LSYLNETVTV SASLESVRGN R**SLFTDLEAE NDVLHCVAFA VPK**SSSNEEV
101 MFLTVQVKGP TQEFKKRTTV MVKNEDSLVF VQTDKSIYKP GQTVKFR**VVS**
151 **MDENFHPLNE LIPLVYIQDP K**GNR**IAQWQS FQLEGGLKQF SFPLSSEPFQ**
201 **GSYK**VVVQKK **SGGRTEHPFT VEEFVLPK**FE VQVTVPKIIT ILEEEMNVSV
251 CGLYTYGKPV PGHVTVSICR KYSDASDCHG EDSQAFCEK**F SGQLNSHGCF**
301 **YQQVK**TKVFQ LKRKEYEMK**L HTEAQIQEEG TVVELTGR**QS SEITRTITKL
351 SFVKVDSHFR QGIPFFGQVR LVDGKGVPIP NKVIFIRGNE ANYYSNATTD
401 EHGLVQFSIN TTNVMGTSLT VRVNYK**DRSP CYGYQWVSEE HEEAHHTAYL**
451 **VFSPSK**SFVH LEPMSHELPC GHTQTVQAHY ILNGGTLLGL KKLSFYYLIM
501 AKGGIVRTGT HGLLVKQEDM KGHFSISIPV KSDIAPVARL LIYAVLPTGD
551 VIGDSAK**YDV ENCLANKVDL SFSPSQSLPA SHAHLR**VTAA PQSVCALRAV
601 DQSVLLMKPD AELSASSVYN LLPEKDLTGF PGPLNDQDNE DCINRHNVYI
651 NGITYTPVSS TNEKDMYSFL EDMGLKAFTN SKIRKPK**MCP QLQQYEMHGP**
701 **EGLR**VGFYES DVMGRGHARL VHVEEPHTET VRKYFPETWI WDLVVVNSAG
751 VAEVGVTVPD TITEWK**AGAF CLSEDAGLGI SSTASLR**AFQ PFFVELTMPY
801 SVIRGEAFTL KATVLNYLPK CIR**VSVQLEA SPAFLAVPVE K**EQAPHCICA
851 NGRQTVSWAV TPKSLGNVNF TVSAEALESQ ELCGTEVPSV PEHGRKDTVI
901 KPLLVEPEGL EK**ETTFNSLL CPSGGEVSEE LSLKLPPNVV EESAR**ASVSV
951 LGDILGSAMQ NTQNLLQMPY GCGEQNMVLF APNIYVLDYL NETQQLTPEI
1001 KSKAIGYLNT GYQRQLNYKH YDGSYSTFGE RYGR**NQGNTW LTAFVLK**TFA
1051 QARAYIFIDE AHITQALIWL SQRQKDNGCF RSSGSLLNNA IKGGVEDEVT
1101 LSAYITIALL EIPLTVTHPV VRNALFCLES AWKTAQEGDH GSHVYTK**ALL**
1151 **AYAFALAGNQ DKR**KEVLKSL NEEAVKKDNS VHWERPQKPK APVGHFYEPQ
1201 APSAEVEMTS YVLLAYLTAQ PAPTSEDLTS ATNIVKWITK **QQNAQGGFSS**
1251 **TQDTVVALHA LSK**YGAATFT RTGKAAQVTI QSSGTFSSKF QVDNNNR**LLL**
1301 **QQVSLPELPG EYSMK**VTGEG CVYLQTSLK**Y NILPEKEEFP FALGVQTLPQ**
1351 **TCDEPKAHTS FQISLSVSYT GSR**ASNMAI VDVKMVSGFI PLKPTVKMLE
1401 RSNHVSRTEV SSNHVLIYLD K**VSNQTLSLF FTVLQDVPVR** DLKPAIVK**VY**
1451 **DYYETDEFAI AEYNAPCSK**D LGNA

Excision iv)

Match to: **CO3_HUMAN** Score: **141** Expect: **1.6e-10**
Complement C3 OS=Homo sapiens GN=C3 PE=1 SV=2
Nominal mass (M_r): **188569**; Calculated pI value: **6.02**
NCBI BLAST search of CO3_HUMAN against nr
Unformatted sequence string for pasting into other applications
Taxonomy: Homo sapiens
Fixed modifications: Carbamidomethyl (C)
Variable modifications: Oxidation (M)
Cleavage by Trypsin: cuts C-term side of KR unless next residue is P
Number of mass values searched: **100**
Number of mass values matched: **37**
Sequence Coverage: **30%**

Matched peptides shown in **Bold Red**

1 MGPTSGPSLL LLLLTHLPLA LGSPMYSIIT PNILRLESEE TMVLEAHDAQ
51 GDVPVTVTVH DFPGKKLVLS SEKTVLTPAT NHMGNVTFTI PANREFKSEK
101 GRNKFVTVQA TFGTQVVEK**V VLVSLQSGYL FIQTDK**TIYT PGSTVLYRIF
151 TVNHKLLPVG RTVMVNIENP EGIPVKQDSL SSQNQLGVLP LSWDIPELVN
201 MGQWKIR**AYY ENSPQQVFST EFEVKEYVLP SFEVIVEPTE K**FYYIYNEKG
251 LEVTITARFL YGK**KVEGTAF VIFGIQDGEQ R**ISLPESLKR IPIEDGSGEV
301 VLSRKVLLDG VQNPRAEDLV GKSLYVSATV ILHSGSDMVQ AER**SGIPIVT**
351 **SPYQIHFTK**T PK**YFKPGMPF DLMVFVTNPD GSPAYR**VPVA VQGEDTVQSL
401 TQGDGVAK**LS INTHPSQKPL SITVR**TKKQE LSEAEQATR**T MQALPYSTVG**
451 **NSNNYLHLSV LRTELRPGET LNVNFLLRMD R**AHEAKIRYY TYLIMNKGRL
501 LKAGRQVR**EP GQDLVVLPLS ITTDFIPSFR LVAYYTLIGA SGQR**EVVADS
551 VWVDVKDSCV GSLVVKSGQS EDRQPVPGQQ MTLKIEGDHG ARVVLVAVDK
601 GVFVLNKKNK LTQSKIWDVV EK**ADIGCTPG SGKDYAGVFS DAGLTFTSSS**
651 **GQQTAQR**AEL QCPQPAARRR RSVQLTEKRM DKVGKYPKEL RKCCEDGMRE
701 NPMRFSCQRR TRFISLGEAC K**KVFLDCCNY ITELRR**QHAR ASHLGLAR**SN**
751 **LDEDIIAEEN IVSRSEFPES WLWNVEDLKE PPK**NGISTKL MNIFLK**DSIT**
801 **TWEILAVSMS DKK**GICVADP FEVTVMQDFF IDLRLPYSVV RNEQVEIRAV
851 LYNYRQNQEL K**VRVELLHNP AFCSLATTK**R RHQQTVTIPP KSSLSVPYVI
901 VPLKTGLQEV EVKAAVYHHF ISDGVRKSLK VVPEGIRMNK TVAVRTLDPE
951 RLGR**EGVQKE DIPPADLSDQ VPDTESETRI LLQGTPVAQM TEDAVDAERL**
1001 **K**HLIVTPSGC GEQNMIGMTP TVIAVHYLDE TEQWEKFGLE KRQGALELIK
1051 KGYTQQLAFR QPSSAFAAFV KRAPSTWLTA YVVKVFSLAV NLIAIDSQVL
1101 CGAVKWLILE K**QKPDGVFQE DAPVIHQEMI GGLR**NNNEKD MALTAFVLIS
1151 LQEAKDICEE QVNSLPGSIT K**AGDFLEANY MNLQRSYTVA IAGYALAQMG**
1201 **R**LKGPLLNKF LTTAKDKNRW EDPGKQLYNV EATSYALLAL LQLKDFDFVP
1251 PVVRWLNEQR YYGGGYGSTQ ATFMVFQALA QYQK**DAPDHQ ELNLDVSLQL**
1301 **PSR**SSKITHR IHWESASLLR SEETKENEGF TVTAEGKGQG TLSVVTMYHA
1351 KAKDQLTCNK FDLKVTIKPA PETEKRPQDA KNTMILEICT RYRGDQDATM
1401 SILDISMMTG FAPDTDDLKQ LANGVDRYIS KYELDKAFSD RNTLIIYLDK
1451 VSHSEDDCLA FK**VHQYFNVE LIQPGAVKVY AYYNLEESCT R**FYHPEKEDG
1501 KLNKLCRDEL CR**CAEENCFI QKSDDK**VTLE ERLDKACEPG VDYVYKTRLV
1551 K**VQLSNDFDE YIMAIEQTIK** SGSDEVQVGQ QRTFISPIKC REALKLEEKK
1601 HYLMWGLSSD FWGEKPNLSY IIGKDTWVEH WPEEDECQDE ENQKQCQDLG
1651 AFTESMVVFG CPN

Match to: **CO4A_HUMAN** Score: **60** Expect: **0.021**
Complement C4-A OS=Homo sapiens GN=C4A PE=1 SV=1
Nominal mass (M_r): **194247**; Calculated pI value: **6.65**
NCBI BLAST search of CO4A_HUMAN against nr
Unformatted sequence string for pasting into other applications
Taxonomy: Homo sapiens
Fixed modifications: Carbamidomethyl (C)
Variable modifications: Oxidation (M)
Cleavage by Trypsin: cuts C-term side of KR unless next residue is P
Number of mass values searched: **100**
Number of mass values matched: **22**
Sequence Coverage: **20%**

Matched peptides shown in **Bold Red**

1 MRLLWGLIWA SSFFTLSLQK PRLLLFSPSV VHLGVPLSVG VQLQDVPRGQ
51 VVKGSVFLRN PSRNNVPCSP KVDFTLSSER **DFALLSLQVP LKDAK**SCGLH
101 QLLRGPEVQL VAHSPWLKDS LSR**TTNIQGI NLLFSSRRGH LFLQTDQPIY**
151 **NPGQRVR**YRV FALDQKMRPS TDTITVMVEN SHGLRVRKKE VYMPSSIFQD
201 DFVIPDISEP GTWKISARFS DGLESNSSTQ FEVKKYVLPN FEVKITPGKP
251 YILTVPGHLD EMQLDIQAR**Y IYGKPVQGVA YVR**FGLLDED GKKTFFRGLE
301 SQTKLVNGQS HISLSKAEFQ DALEKLNMGI TDLQGLRLYV AAAIIESPGG
351 EMEEAELTSW YFVSSPFSLD LSKTKR**HLVP GAPFLLQALV R**EMSGSPASG
401 IPVKVSATVS SPGSVPEVQD IQQNTDGSGQ VSIPIIIPQT ISELQLSVSA
451 GSPHPAIAR**L TVAAPPSGGP GFLSIERPDS RPPR**VGDTLN LNLR**AVGSGA**
501 **TFSHYYYMIL SR**GQIVFMNR EPKRTLTSVS VFVDHHLAPS FYFVAFYYHG
551 DHPVANSLRV DVQAGACEGK LELSVDGAKQ YRNGESVKLH LETDSLALVA
601 LGALDTALYA AGSKSHKPLN MGKVFEAMNS YDLGCGPGGG DSALQVFQAA
651 GLAFSDGDQW TLSRKRLSCP KEKTTRKKRN VNFQKAINEK LGQYASPTAK

701 RCCQDGVTRL PMMRSCEQRA AR**VQQPDCRE PFLSCCQFAE SLR**KKSRDKG
751 QAGLQR**ALEI LQEEDLIDED DIPVR**SFFPE NWLWRVETVD RFQILTLWLP
801 DSLTTWEIHG LSLSKTKGLC VATPVQLRVF REFHLHLRLP MSVRRFEQLE
851 LRPVLYNYLD KNLTVSVHVS PVEGLCLAGG GGLAQQVLVP AGSARPVAFS
901 VVPTAAAAVS LKVVARGSFE FPVGDAVSKV LQIEK**EGAIH REELVYELNP**
951 **LDHR**GR**TLEI PGNSDPNMIP DGDFNSYVRV TASDPLDTLG SEGALSPGGV**
1001 **ASLLR**LPRGC GEQTMIYLAP TLAASRYLDK TEQWSTLPPE TKDHAVDLIQ
1051 KGYMRIQQFR KADGSYAAWL SRDSSTWLTA FVLKVLSLAQ EQVGGSPEKL
1101 QETSNWLLSQ QQADGSFQDP CPVLDRSMQG GLVGNDETVA LTAFVTIALH
1151 HGLAVFQDEG AEPLKQRVEA SISKANSFLG EKASAGLLGA HAAAITAYAL
1201 SLTKAPVDLL GVAHNNLMAM AQETGDNLYW GSVTGSQSNA VSPTPAPRNP
1251 SDPMPQAPAL WIETTAYALL HLLLHEGK**AE MADQASAWLT RQGSFQGGFR**
1301 **STQDTVIALD ALSAYWIASH TTEER**GLNVT LSSTGRNGFK SHALQLNNRQ
1351 IR**GLEEELQF SLGSKINVK**V GGNSKGTLKV LRTYNVLDMK NTTCQDLQIE
1401 VTVKGHVEYT MEANEDYEDY EYDELPAKDD PDAPLQPVTP LQLFEGRRNR
1451 RRREAPKVVE EQESRVHYTV CIWRNGKVGL SGMAIADVTL LSGFHALRAD
1501 LEKLTSLSDR **YVSHFETEGP HVLLYFDSVP TSR**ECVGFEA VQEVPVGLVQ
1551 PASATLYDYY NPERRCSVFY GAPSKSR**LLA TLCSAEVCQC AEGK**CPRQRR
1601 ALERGLQDED GYRMKFACYY PRVEYGFQVK VLREDSRAAF RLFETKITQV
1651 LHFTKDVKAA ANQMRNFLVR ASCRLRLEPG KEYLIMGLDG ATYDLEGHPQ
1701 YLLDSNSWIE EMPSERLCRS TRQR**AACAQL NDFLQEYGTQ GCQV**

Match to: **CO4B_HUMAN** Score: **68** Expect: **0.0036**
Complement C4-B OS=Homo sapiens GN=C4B PE=1 SV=1
Nominal mass (M_r): **194212**; Calculated pI value: **6.73**
NCBI BLAST search of CO4B_HUMAN against nr
Unformatted sequence string for pasting into other applications
Taxonomy: Homo sapiens
Fixed modifications: Carbamidomethyl (C)
Variable modifications: Oxidation (M)
Cleavage by Trypsin: cuts C-term side of KR unless next residue is P
Number of mass values searched: **100**
Number of mass values matched: **23**
Sequence Coverage: **21%**
Matched peptides shown in **Bold Red**

1 MRLLWGLIWA SSFFTLSLQK PRLLLFSPSV VHLGVPLSVG VQLQDVPRGQ
51 VVKGSVFLRN PSRNNVPCSP KVDFTLSSER **DFALLSLQVP LKDAK**SCGLH
101 QLLRGPEVQL VAHSPWLKDS LSR**TTNIQGI NLLFSSRRGH LFLQTDQPIY**
151 **NPGQRVR**YRV FALDQKMRPS TDTITVMVEN SHGLRVRKKE VYMPSSIFQD
201 DFVIPDISEP GTWKISARFS DGLESNSSTQ FEVKKYVLPN FEVKITPGKP
251 YILTVPGHLD EMQLDIQAR**Y IYGKPVQGVA YVR**FGLLDED GKKTFFRGLE
301 SQTKLVNGQS HISLSKAEFQ DALEKLNMGI TDLQGLRLYV AAAIIESPGG
351 EMEEAELTSW YFVSSPFSLD LSKTKR**HLVP GAPFLLQALV R**EMSGSPASG
401 IPVKVSATVS SPGSVPEVQD IQQNTDGSGQ VSIPIIIPQT ISELQLSVSA
451 GSPHPAIAR**L TVAAPPSGGP GFLSIERPDS RPPR**VGDTLN LNLR**AVGSGA**
501 **TFSHYYYMIL SR**GQIVFMNR EPKRTLTSVS VFVDHHLAPS FYFVAFYYHG
551 DHPVANSLRV DVQAGACEGK LELSVDGAKQ YRNGESVKLH LETDSLALVA
601 LGALDTALYA AGSKSHKPLN MGKVFEAMNS YDLGCGPGGG DSALQVFQAA
651 GLAFSDGDQW TLSRKRLSCP KEKTTRKKRN VNFQKAINEK LGQYASPTAK
701 RCCQDGVTRL PMMRSCEQRA AR**VQQPDCRE PFLSCCQFAE SLR**KKSRDKG
751 QAGLQR**ALEI LQEEDLIDED DIPVR**SFFPE NWLWRVETVD RFQILTLWLP
801 DSLTTWEIHG LSLSKTKGLC VATPVQLRVF REFHLHLRLP MSVRRFEQLE
851 LRPVLYNYLD KNLTVSVHVS PVEGLCLAGG GGLAQQVLVP AGSARPVAFS
901 VVPTAAAAVS LKVVARGSFE FPVGDAVSKV LQIEK**EGAIH REELVYELNP**
951 **LDHR**GR**TLEI PGNSDPNMIP DGDFNSYVRV TASDPLDTLG SEGALSPGGV**
1001 **ASLLR**LPRGC GEQTMIYLAP TLAASRYLDK TEQWSTLPPE TKDHAVDLIQ
1051 KGYMRIQQFR KADGSYAAWL SRDSSTWLTA FVLKVLSLAQ EQVGGSPEK**L**
1101 **QETSNWLLSQ QQADGSFQDL SPVIHR**SMQG GLVGNDETVA LTAFVTIALH
1151 HGLAVFQDEG AEPLKQRVEA SISKANSFLG EKASAGLLGA HAAAITAYAL
1201 SLTKAPVDLL GVAHNNLMAM AQETGDNLYW GSVTGSQSNA VSPTPAPRNP
1251 SDPMPQAPAL WIETTAYALL HLLLHEGK**AE MADQASAWLT RQGSFQGGFR**
1301 **STQDTVIALD ALSAYWIASH TTEER**GLNVT LSSTGRNGFK SHALQLNNRQ
1351 IR**GLEEELQF SLGSKINVK**V GGNSKGTLKV LRTYNVLDMK NTTCQDLQIE
1401 VTVKGHVEYT MEANEDYEDY EYDELPAKDD PDAPLQPVTP LQLFEGRRNR
1451 RRREAPKVVE EQESRVHYTV CIWRNGKVGL SGMAIADVTL LSGFHALRAD
1501 LEKLTSLSDR **YVSHFETEGP HVLLYFDSVP TSR**ECVGFEA VQEVPVGLVQ

1551 PASATLYDYY NPERRCSVFY GAPSKSR**LLA TLCSAEVCQC AEGK**CPRQRR
1601 ALERGLQDED GYRMKFACYY PRVEYGFQVK VLREDSRAAF RLFETKITQV
1651 LHFTKDVKAA ANQMRNFLVR ASCRLRLEPG KEYLIMGLDG ATYDLEGHPQ
1701 YLLDSNSWIE EMPSERLCRS TRQR**AACAQL NDFLQEYGTQ GCQV**

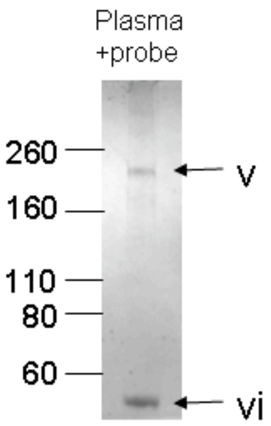

Excision vi)

Match to: **ALBU_HUMAN** Score: **153** Expect: **1e-11**
Serum albumin OS=Homo sapiens GN=ALB PE=1 SV=2
Nominal mass (M_r): **71317**; Calculated pI value: **5.92**
NCBI BLAST search of ALBU_HUMAN against nr
Unformatted sequence string for pasting into other applications
Taxonomy: Homo sapiens
Fixed modifications: Carbamidomethyl (C)
Variable modifications: Oxidation (M)
Cleavage by Trypsin: cuts C-term side of KR unless next residue is P
Number of mass values searched: **79**
Number of mass values matched: **21**
Sequence Coverage: **47%**

Matched peptides shown in **Bold Red**

1 MKWVTFISLL FLFSSAYSRG VFRRDAHKSE VAHRFKDLGE ENFK**ALVLIA**
51 **FAQYLQQCPF EDHVK**LVNEV TEFAKTCVAD ESAENCDK**SL HTLFGDKLCT**
101 **VATLR**ETYGE MADCCAKQEP ERNECFLQHK DDNPNLPR**LV RPEVDVMCTA**
151 **FHDNEETFLK K**YLYEIARR**H PYFYAPELLF FAKR**YK**AAFT ECCQAADKAA**
201 **CLLPK**LDELR DEGKASSAKQ RLKCASLQKF GERAFKAWAV ARLSQRFPKA
251 EFAEVSKLVT DLTK**VHTECC HGDLLECADD RADLAK**YICE NQDSISSKLK
301 ECCEKPLLEK **SHCIAEVEND EMPADLPSLA ADFVESK**DVC K**NYAEAKDVF**
351 **LGMFLYEYAR** R**HPDYSVVLL LRLAK**TYETT LEKCCAAADP HECYAK**VFDE**
401 **FKPLVEEPQN LIKQNCELFE QLGEYKFQNA LLVR**YTK**KVP QVSTPTLVEV**
451 **SR**NLGKVGSK CCKHPEAK**RM PCAEDYLSVV LNQLCVLHEK** TPVSDRVTKC
501 CTESLVNR**RP CFSALEVDET YVPKEFNAET FTFHADICTL SEKER**QIKKQ
551 TALVELVKHK PKATKEQLKA VMDDFAAFVE KCCKADDKET CFAEEGKKLV
601 AASQAALGL
